# A Comparison of Monoaxial Pedicle Screws Versus Polyaxial Pedicle Screws in Posterior Fusion Surgery for Adolescent Idiopathic Scoliosis (Lenke Type 1)

**DOI:** 10.7759/cureus.107673

**Published:** 2026-04-24

**Authors:** Ahmed Hammad, Mohamed E Saleh, Abd-Elfatah A Shehab

**Affiliations:** 1 Neurosurgery, Faculty of Medicine, Al-Azhar University, Assiut, EGY

**Keywords:** adolescent idiopathic scoliosis, mono-axial, pedicle screws, poly-axial, posterior fusion

## Abstract

Background

Adolescent idiopathic scoliosis (AIS) is the most common form of spinal deformity in adolescents, characterized by a three-dimensional curvature of the spine with unknown etiology. Surgical intervention is indicated in progressive curves, particularly in Lenke type 1 patterns, to achieve deformity correction, spinal balance, and long-term stability.

Objective

The objective of this study is to compare the clinical and radiological outcomes of mono-axial versus poly-axial pedicle screw constructs in posterior spinal fusion surgery for AIS Lenke type 1, with particular emphasis on deformity correction, and operative parameters.

Patients and methods

This hospital-based prospective comparative study was conducted at the Faculty of Medicine, Al-Azhar University Hospital, Assiut. The study included 30 patients with AIS who attended the outpatient clinic and were indicated for posterior scoliosis correction surgery during the study period. Patients were enrolled after fulfilling the inclusion criteria and were divided into two groups according to the type of pedicle screw instrumentation used. All patients were evaluated and compared regarding demographic data (age, sex), clinical presentation, radiological parameters, operative details, and postoperative outcomes.

Results

This study included 30 patients with AIS Lenke type 1, divided equally into mono-axial and poly-axial pedicle screw groups (n=15 each). The mean ages were 13.87 years in the monoaxial group and 14.33 years in the polyaxial group with no statistical differences (p=0.386). Women represented 93.3% of patients in both groups, with no statistically significant difference in gender distribution (p=1.000, χ²=0.00).

There was no statistically significant difference between the two groups regarding operative duration (p=0.907) or intraoperative blood loss (p=0.411). Radiological outcomes showed significant postoperative improvement in both groups; however, no statistically significant difference was found between mono-axial and poly-axial screws in preoperative or postoperative major Cobb angle (p=0.439 and p=0.670, respectively), apical vertebral translation (p=0.966 preoperatively and p=0.501 postoperatively), or T1-S1 spinal height (p=0.884 preoperatively and p=0.874 postoperatively). VAS and Oswestry Disability Index improved postoperatively in both groups, with no statistically significant difference between the examined groups (p>0.05).

Conclusion

In patients with moderate Lenke type 1 AIS treated with posterior segmental pedicle screw instrumentation and rod derotation, monoaxial and polyaxial pedicle screw constructs produced comparable radiographic correction and clinical outcomes in our series.

## Introduction

Scoliosis is a spinal deformity characterized by a lateral curvature greater than 10°, associated with vertebral rotation and abnormalities in the sagittal plane [1]. Adolescent idiopathic scoliosis (AIS) is a three-dimensional spinal deformity of unknown etiology and accounts for approximately 85% of all scoliosis cases [1]. It typically presents during adolescence and has a reported prevalence of 1%-3% in the general population, with a higher incidence and greater risk of progression in women, particularly when the curve magnitude exceeds 40° [1].

AIS is commonly classified according to the age of onset into infantile, juvenile, and adolescent types, with adolescent-onset scoliosis being the most prevalent [1,2]. The deformity most frequently presents as a right thoracic curve accompanied by a compensatory left lumbar curve [1,2]. Although the precise etiology remains unclear, several factors have been proposed, including genetic predisposition, hormonal influences, neuromuscular imbalance, and vestibular dysfunction [2].

Clinical evaluation involves careful inspection for trunk asymmetry and the use of the Adam’s forward bending test to identify structural spinal curvature [1]. A comprehensive neurological examination is essential to exclude underlying neurological pathology [1]. Radiological assessment using standing posteroanterior and lateral spinal radiographs remains fundamental for measuring the Cobb angle, classifying the deformity, assessing skeletal maturity, and guiding surgical planning [1].

Surgical treatment is generally indicated for progressive curves to prevent further deformity progression, restore spinal alignment, and improve cosmetic appearance [1,3]. Posterior spinal fusion with pedicle screw instrumentation has become the standard surgical approach for AIS because it allows effective three-dimensional correction while maintaining spinal balance and providing stable fixation for fusion [1,3]. Advances in implant design and corrective techniques have further enhanced surgical outcomes [1,3].

Despite advances in posterior segmental pedicle screw instrumentation and rod derotation techniques in AIS, there is still ongoing debate regarding the optimal type of pedicle screw fixation. Monoaxial screws provide strong corrective forces through controlled rod derotation, whereas polyaxial screws offer greater flexibility in rod placement and ease of insertion. However, limited evidence exists comparing their clinical and radiological outcomes specifically in Lenke type 1 deformities.

## Materials and methods

Study design

This prospective study was conducted on 30 patients who presented to the outpatient clinic and were indicated for surgical correction of scoliosis. Patients were divided into two groups according to the pedicle screw technique used.

Inclusion criteria

Patients were included if they met the following criteria: (a) Diagnosis of adolescent idiopathic scoliosis (AIS); (b) age between 10 and 18 years; (c) Lenke type 1 deformity; (d) right-sided thoracic curve with compensatory left lumbar curve; (e) Cobb angle between 40° and 80°; (f) foth male and female patients; and (g) willingness to participate in the study and provide informed consent.

Exclusion criteria

Patients were excluded if they had: (a) non-idiopathic scoliosis; (b) age below 10 years or above 18 years; (c) left-sided thoracic curve; (d) Cobb angle less than 40°; (e) spinal infection; and (f) history of previous spinal surgery.

Sample size

A total of 30 patients were included in the study after obtaining informed consent from each participant.

Sampling method and randomization

Thirty patients who fulfilled the inclusion criteria were randomly assigned to either study group. Randomization was performed using a computer-generated randomization sheet using MedCalc® software version 19 (MedCalc Software Ltd, Ostend, Belgium).

Ethical considerations

Written informed consent was obtained from all patients and their guardians after providing a clear explanation regarding the nature, purpose, and potential risks of the study. Patient confidentiality was maintained by recording only patient initials in the study database, while a separate list linking patient names and initials was securely kept by the researchers. Ethical approval was obtained from the Neurosurgery Department Council at Al-Azhar University before the initiation of the study, in accordance with local ethical regulations. Patients were informed about potential complications related to spinal surgery, including bleeding, infection, postoperative pain, and shoulder imbalance.

Preoperative evaluation

All patients underwent a comprehensive clinical assessment, including detailed history taking, physical examination, neurological examination, and radiological evaluation. Clinical assessment included evaluation of overall appearance, spinal deformity, skin findings suggestive of neurocutaneous syndromes, neuromuscular status, and height measurement to monitor skeletal growth and curve progression.

A detailed neurological examination was performed to assess coronal and sagittal balance, motor power, sensory function, gait pattern, deep tendon reflexes, and the presence of pathological reflexes such as the Babinski sign. Radiological assessment included standing anteroposterior and lateral spinal radiographs for Cobb angle measurement and deformity assessment. Dynamic radiographs were obtained to evaluate curve flexibility. MRI of the spine was performed to identify intraspinal abnormalities, while CT scan of the whole spine was obtained when indicated. Routine laboratory investigations and preoperative pulmonary function tests were performed for all patients.

All patients underwent full preoperative preparation, including cardiology and pulmonology consultations and anesthetic evaluation. Patients and their families received detailed counseling regarding the surgical procedure, expected outcomes, possible complications, and postoperative rehabilitation.

Surgical technique

After induction of general anesthesia, patients were positioned prone on bolsters with appropriate padding of pressure points and head support. Neuromonitoring electrodes were applied and baseline recordings were obtained prior to the start of surgery. When necessary, a radiolucent operating table was used to facilitate intraoperative imaging.

A midline posterior incision was performed, followed by subperiosteal exposure of the posterior spinal elements to identify anatomical landmarks and prepare the fusion bed. Pedicle screws were inserted using the freehand technique guided by anatomical landmarks. Screw placement was verified using a ball-tipped probe and triggered electromyography when indicated.

Correction maneuvers included soft tissue releases and spinal osteotomies depending on the severity and rigidity of the curve. Ponte osteotomies were performed in moderate curves, whereas more extensive osteotomies such as pedicle subtraction osteotomy or vertebral column resection were reserved for severe or rigid deformities. Thoracoplasty was performed in selected cases for rib hump correction.

Following deformity correction, rods were secured to the screws and final tightening was performed. The wound was then closed in layers. Postoperative management included adequate analgesia, respiratory exercises, neuromonitoring observation, and early mobilization as part of the rehabilitation protocol.

Study groups

The study included 30 patients who were divided into two groups according to the pedicle screw system used: Group A: Posterior spinal fusion using monoaxial pedicle screws (n=15); Group B: Posterior spinal fusion using polyaxial pedicle screws (n=15).

Questionnaires/scores

Pain intensity was assessed using the Visual Analog Scale (VAS), a validated tool widely used for the measurement of pain intensity in clinical practice [4]. The scale consists of a 10-cm horizontal line representing a continuum from “no pain” to “worst imaginable pain,” where patients mark the point that best reflects their perceived pain level.

Functional disability was evaluated using the Oswestry Disability Index (ODI), a standardized questionnaire designed to assess disability related to spinal disorders [5]. The ODI includes 10 sections covering pain intensity and daily activities such as personal care, lifting, walking, sitting, standing, sleeping, social life, traveling, and employment. Scores range from 0 to 100, with higher scores indicating greater functional disability.

## Results

Table 1 summarizes the demographic, operative, radiological, and clinical data of the included patients. The mean ages were 13.87 years in the monoaxial group and 14.33 years in the polyaxial group, with no statistically significant difference (p=0.386). The majority of the included patients were women (n=14, 93.3%) in each group, with no statistically significant difference between the two groups (p=1.000, χ²=0.00).

**Table 1:** Demographic, operative, radiological, and clinical data of the included patients *Mean (SD), ^#^Median (IQR). SD: Standard deviation; IQR: interquartile range. min: minutes, ml: milliliters, mm: millimeters, AVT: Apical Vertebral Translation, T1-S1: Distance from the first thoracic vertebra to the first sacral vertebra, ODI: Oswestry Disability Index, %: percentage.

Variables		Group A	Group B	P value	Student’s t test
Gender	Male	1 (6.7%)	1 (6.7%)	1.000	-
	Female	14 (93.3%)	14 (93.3%)		
Age		13.87 (1.356)*	14.33 (1.543)*	0.386	-0.8799
Duration of operation (min)		261.67 (19.70)*	262.67 (26.31)*	0.907	-0.1178
Intraoperative estimated blood loss (ml)		594.67 (58.42)*	615.33 (76.05)*	0.411	-0.8347
Major cobb angle preoperative		60.60 (5.07)*	62.20 (6.06)*	0.439	-0.7843
Major cobb angle postoperative		17.73 (2.99)*	18.20 (2.96)*	0.670	-0.4300
Apical vertebral translation preoperative (mm)		46.53 (3.93)*	46.60 (4.53)*	0.966	-0.0431
Apical vertebral translation postoperative (mm)		18.40 (1.96)*	18.93 (2.31)*	0.501	-0.6817
T1-S1 height preoperative (mm)		349.00 (5.10)*	348.73 (4.83)*	0.884	0.1470
T1-S1 height postoperative (mm)		386.73 (4.62)*	386.47 (4.50)*	0.874	0.1601
Oswestry disability index (%)		12.07 (1.58)*	12.20 (2.11)*	0.846	-0.196
Visual analog scale preoperative		6 (1)^#^	6 (1)^#^	0.806	-
Visual analog scale postoperative		2 (1)^#^	2 (1)^#^	0.735	-

The operative data showed that the mean operative duration (in minutes) did not differ significantly between the monoaxial and polyaxial groups (p=0.907). Similarly, the intraoperative estimated blood loss (in milliliters) was comparable between the two groups with no statistically significant difference (p=0.411).

Regarding the radiological parameters, the major Cobb angle showed no statistically significant difference between the monoaxial and polyaxial groups in the preoperative values (p=0.439) or in the postoperative values (p=0.670). The apical vertebral translation also showed no statistically significant difference between the two groups in the preoperative measurements (p=0.966) or the postoperative measurements (p = 0.501). Likewise, the T1-S1 spinal height demonstrated no statistically significant difference between the two groups in the preoperative measurements (p = 0.884) or the postoperative measurements (p=0.874).

The visual analog scale (VAS) was comparable between the monoaxial and polyaxial groups in both the preoperative (p=0.806) and postoperative assessments (p=0.735). Similarly, the ODI % showed no statistically significant difference between the two groups in the preoperative scores (p = 0.846).

## Discussion

Since the introduction of Harrington instrumentation in the early 1960s [6], techniques aimed at three-dimensional correction of AIS have evolved substantially. Rod derotation (RD) and posterior segmental pedicle screw instrumentation (PSI) have become central to modern posterior corrective strategies, providing powerful corrective forces transmitted from contoured rods through pedicle screws to the vertebrae [6]. These concepts laid the groundwork for current posterior-only strategies that allow substantial coronal, sagittal, and axial correction while often minimizing fused levels.

In this study, we compared monoaxial and polyaxial pedicle screw constructs in posterior fusion for Lenke type 1 AIS and found no statistically significant differences in demographic variables, operative parameters (operative time and estimated blood loss), radiographic correction (pre- and postoperative major Cobb angle, apical vertebral translation, and T1-S1 height), or patient-reported outcomes (VAS and ODI). In short, both constructs achieved comparable deformity correction and clinical improvement in patients with moderate main thoracic curves.

Our results corroborate earlier comparative clinical reports that found broadly equivalent coronal correction between monoaxial and multiaxial/polyaxial screw constructs, while differences - when reported - tended to be limited to measures of apical derotation or thoracic symmetry rather than global coronal correction. For example, Kuklo et al. compared monoaxial versus multiaxial thoracic pedicle screws for correction of Lenke I AIS and concluded that both screw types produced excellent coronal deformity correction; however, monoaxial screws produced superior measures of derotation and thoracic symmetry in their cohort [7].

Other clinical studies report similar nuances. Dalal and colleagues found that polyaxial (multiaxial) constructs allow sufficient coronal correction but that monoaxial/uniplanar constructs may produce greater apical vertebral rotation correction in some series [8]. Lonner and co-workers noted that polyaxial constructs were associated with better maintenance of thoracic kyphosis compared with monoaxial or hybrid constructs, which showed a trend toward kyphosis loss; this observation highlights that the effect of screw type may be parameter-specific (i.e., affecting rotation or sagittal contours more than coronal Cobb correction) [9].

Taken together, the clinical literature indicates that while screw head design has measurable biomechanical differences, these do not uniformly translate into large, clinically meaningful differences in overall coronal curve correction for many AIS patients, particularly in cohorts with moderate curves treated via posterior-only segmental fixation [10].

Biomechanical investigations demonstrate differences in force transmission and local stresses between rigid (monoaxial/uniplanar) and flexible (polyaxial/multiaxial) screw heads [10]. Monoaxial screws, with a fixed head, provide a rigid linkage that can theoretically transmit rotational moments more directly to the vertebral body, which may increase apical derotation but also concentrates stress at the bone-screw interface [10]. Polyaxial screws, with a mobile head, are more forgiving during rod seating and may distribute forces more gradually, reducing focal pedicle strain and potentially lowering the risk of screw failure in certain bone qualities or complex maneuvers [10]. Experimental studies of screw-head design in derotation maneuvers and screw purchase mechanics describe these trade-offs and support the notion that the final clinical effect is determined by a complex interaction of construct design, rod contouring, surgical technique, and patient anatomy [10].

A crucial point that helps reconcile discordant findings between biomechanical expectations and clinical results is surgical technique. Techniques such as combined rod-and-screw-head derotation (applying corrective forces using both the rod and the screw head) or direct vertebral rotation (DVR) can modify how corrective moments are transmitted to the spine and can compensate for the theoretical disadvantages of a given screw head design [11]. In our series, we routinely applied a combined rod-and-screw-head derotation maneuver (as described in recent technique literature), which likely reduced the practical difference between monoaxial and polyaxial constructs during RD and may explain the equivalent radiographic outcomes observed.

Derotational maneuvers and posteromedial translation that aim to correct axial deformity can, by their mechanical nature, also influence sagittal alignment and create residual coronal or axial asymmetries if not properly controlled. Recent multicenter and methodological analyses have cautioned that derotation-focused strategies may increase thoracolumbar lordosis or create focal sagittal changes that could predispose to junctional problems if not anticipated; likewise, posteromedialization may leave some residual axial deformity depending on the plane of applied forces. These considerations underline that technique selection and attention to sagittal balance are as important as implant choice [8].

Patient-reported outcomes (pain, function) after AIS surgery are multifactorial. Several studies suggest that factors such as fusion length, construct stiffness, and complications (screw loosening, pseudarthrosis) exert a greater influence on long-term pain and disability than the nominal flexibility of the screw head itself [12]. In our cohort, the VAS and ODI improved similarly across groups, concordant with prior reports that pedicle screw fixation in pediatric patients generally yields good pain and functional results and that screw head type alone is a weak predictor of patient-reported outcome. Nevertheless, complications related to pedicle screws (malposition, loosening, neurologic or vascular injury) remain important considerations, and the risk profile must be weighed when choosing instrumentation strategies.

Given comparable coronal correction and clinical outcomes in our study, implant selection can reasonably prioritize practical intraoperative considerations: ease of rod seating, need for incremental adjustments, surgeon familiarity, implant inventory, and cost. Polyaxial screws may facilitate rod insertion and reduce operative difficulty in stiff curves or when multiple contour adjustments are anticipated, while monoaxial screws may be favored in cases where maximal uniplanar control and direct transmission of rotational moments are desired - but the clinical payoff for overall deformity correction appears limited in many moderate AIS cases when modern derotation techniques are applied [12].

This study has limitations that temper broad generalization. It includes a relatively small sample size, which reduces statistical power to detect subtle differences in some radiographic or clinical endpoints and increases susceptibility to selection bias. Follow-up duration is limited, so we cannot comment on the long-term durability of corrections, implant longevity, or late complications such as junctional failure or late implant loosening. Additionally, despite efforts to standardize surgical technique, surgeon preference and small technique variations (e.g., sequence of correction, use of osteotomies, rod contour and stiffness) may influence outcomes. Finally, although we compared common radiographic and clinical metrics, more sensitive measures of axial rotation (CT-based rotation indices) or 3D reconstructions might reveal small differences not captured by standard plain radiographs. Future prospective, multicenter studies with larger cohorts, longer follow-up, and standardized operative protocols (or randomized implant allocation where feasible) would provide stronger evidence on whether any clinically important advantage exists for either screw type. Recent comparative analyses and systematic reviews call for such larger, higher-quality studies.

## Conclusions

In patients with moderate Lenke type 1 AIS treated with posterior segmental pedicle screw instrumentation and rod derotation, monoaxial and polyaxial screws showed comparable radiographic and clinical outcomes. Although biomechanical differences exist, their practical impact appears minimal with modern derotation techniques. Implant selection should therefore depend on surgeon preference and case-specific factors.
